# Progress in studying the impact of hyperlipidemia and statins on rotator cuff injury and repair

**DOI:** 10.3389/fpubh.2023.1279118

**Published:** 2023-10-27

**Authors:** Yinhua Qian, Haoqiang Huang, Renwen Wan, Yu Zhou, Xinting Feng, Feng Xu, Zhiwen Luo, Qing Wang

**Affiliations:** ^1^Department of Orthopaedics, Kunshan Hospital of Traditional Chinese Medicine, Kunshan, Jiangsu, China; ^2^Department of Sports Medicine, Huashan Hospital, Fudan University, Shanghai, China

**Keywords:** rotator cuff injury, hyperlipidemia, statin, mechanism, treatment, inflammation

## Abstract

This review delves into the intersection of two prevalent conditions, hyperlipidemia and rotator cuff injuries, both of which bear substantial healthcare burdens. Our investigation begins with an exploration of rotator cuff injuries, common musculoskeletal disorders that severely impair shoulder functionality and quality of life. These injuries are notably pervasive among sports enthusiasts and the older adult, with an incidence rate estimated at 5–10% in the general population. Despite their widespread occurrence and the diverse, multifactorial etiological factors, effective treatment strategies remain elusive. We then examine hyperlipidemia, a metabolic disorder affecting approximately 40% of the global adult population. Characterized by elevated levels of cholesterol and triglycerides, hyperlipidemia can precipitate severe cardiovascular complications and presents a significant socioeconomic burden. Although current management strategies encompass lifestyle modifications and pharmacological interventions, the condition remains a formidable health challenge. Central to this review is the exploration of a potential association between hyperlipidemia and rotator cuff injuries. We aim to synthesize the current understanding of hyperlipidemia’s role in the pathophysiology of rotator cuff injuries, thereby offering fresh insights into their common etiological underpinnings, potential therapeutic targets, and drugs, such as Statins. The influence of other lipid-lowering therapeutics on tendon health is also considered, and further research into the molecular pathways and potential therapeutic benefits of these drugs is required. This pursuit aligns with broader efforts to enhance patient outcomes, minimize healthcare burdens, and contribute to the global understanding of these prevalent conditions.

## Introduction

The rotator cuff, a sophisticated orchestration of muscles and tendons that envelope the shoulder joint, serves an indispensable role in bestowing stability and enabling a diverse array of shoulder movements (1–3). Afflictions to this vital assembly, which span the spectrum from benign inflammation or tendonitis to partial or even full-throttle tendon ruptures, predominantly impact the shoulder’s soft tissues (4, 5). These injuries frequently culminate in acute pain, muscular weakness, and restricted mobility, thereby epitomizing a pervasive musculoskeletal disorder (6). Rotator cuff injuries manifest with particular frequency among sports aficionados and the geriatric population, often inducing substantial debilitation in shoulder functionality and a significant deterioration in the quality of life (3). The annual incidence rate of rotator cuff injuries hovers around 5–10% within the general populace, with a conspicuous surge in prevalence commensurate with advancing age (asymptomatic tears were found in more than 20% of individuals over the age of 60) (7). A rough estimate of the economic burden for a single patient undergoing surgery for a rotator cuff injury can be in the range of $10,000–$30,000 or even more, including both direct and indirect costs. Thus, this condition represents a substantial quota of visits to orthopedic clinics, exerting a hefty strain on healthcare resources, and thereby emerging as a noteworthy public health quandary.

The precise etiology of rotator cuff injuries remains nebulous, likely being multifactorial in nature. The onslaught of years is a well-entrenched risk factor, with degenerative alterations in the rotator cuff tendons becoming increasingly commonplace as individuals age (8). Furthermore, lifestyle determinants such as smoking have been implicated, with empirical evidence indicating that smokers are at an escalated risk of succumbing to rotator cuff injuries in contrast to their non-smoking counterparts (9). Medical conditions such as diabetes and hypertension have also been correlated with an augmented risk of these injuries, thereby suggesting a role for systemic health in the integrity of the rotator cuff (10, 11). The stewardship of rotator cuff injuries is multifarious, with therapeutic options customized to the individual patient’s needs, factoring in the severity of the injury, the patient’s overarching health status, and their functional exigencies. Conservative management approaches, encompassing physical therapy, pain mitigation with non-steroidal anti-inflammatory drugs (NSAIDs), and corticosteroid injections, typically constitute the initial line of treatment (12). However, in cases of severe injuries or when conservative management proves ineffective, surgical intervention may be necessitated (8). Despite these treatment modalities, a significant number of patients persist in experiencing pain and functional limitations, thereby highlighting the need for further research into innovative therapeutic strategies (11, 13).

Hyperlipidemia, a pervasive metabolic aberration, is characterized by escalated levels of total cholesterol (TC), triglycerides (TG), and low-density lipoprotein cholesterol (LDL-C), coupled with a reduction in high-density lipoprotein cholesterol (HDL-C) (1, 14, 15). This condition often manifests covertly, with no symptoms in its incipient stages. However, unaddressed hyperlipidemia can precipitate atherosclerosis, engendering grave cardiovascular ramifications such as heart disease, cerebrovascular accident, and peripheral artery disease (10, 16, 17). As a substantial global health quandary, the World Health Organization posits that approximately 40% of the global adult populace grapples with elevated total cholesterol levels, a key indicator of hyperlipidemia. This widespread disorder imposes a significant socioeconomic encumbrance, accounting for a considerable proportion of healthcare expenditure. Therapeutic stratagems encompass lifestyle modifications, including dietary adjustments and consistent physical exertion, along with pharmacological interventions like statins, fibrates, and niacin (16, 18, 19). Despite these measures, hyperlipidemia persists as a formidable health challenge, underscoring the exigency for continued exploration into innovative therapeutic approaches and prophylactic strategies. The potential correlation between hyperlipidemia and rotator cuff injuries further accentuates the necessity for a comprehensive understanding and efficacious management of this disorder (16).

Given the substantial prevalence and socio-economic burden of hyperlipidemia (20, 21), and its potential association with rotator cuff injuries, a comprehensive understanding of the interplay between these two conditions is essential. Therefore, this review aims to amalgamate the current understanding of the role of hyperlipidemia in the pathophysiology of rotator cuff injuries. Our objective is to elucidate this connection, thereby providing novel insights for etiological research and clinical management. By probing into the underlying mechanisms, we expect to shed light on potential therapeutic targets and preventive strategies, thereby contributing to the global efforts in addressing these prevalent conditions.

## Hyperlipidemia as a risk factor for rotator cuff injuries

The etiology of rotator cuff injuries is a complex interplay of multiple factors, inclusive of age-related degenerative changes, disease progression, and undue physical strain. In the scientific discourse of recent years, a considerable emphasis has been placed on the potential correlation between hyperlipidemia and rotator cuff injuries. This connection was evidenced in a prospectively designed study by Skovgaard et al. (16), wherein hypercholesterolemia surfaced as a factor escalating the risk of upper limb tendon injuries by a factor of 1.5. A similar correlation was delineated in Kara’s research (10), where cardiovascular risk elements, including diabetes and hyperlipidemia, were identified as potential precipitators of rotator cuff injuries.

In a parallel vein, Djerbi et al. (22) uncovered a significant correlation between smoking, dyslipidemia, and the incidence rate of rotator cuff tears. An 11-year longitudinal follow-up study conducted by Lin et al. (15) further underscored hyperlipidemia as an independent risk factor for rotator cuff disorders, doubling the risk compared to non-hyperlipidemic cohorts. Recent studies echoed these findings, demonstrating a discernible link between hyperlipidemia and various manifestations of rotator cuff tendon diseases, with hyperlipidemic patients bearing a higher risk of complete tears.

However, it is imperative to note the existence of contrarian views, as several studies (23, 24) reported no significant correlation between dyslipidemia and rotator cuff tears. These incongruities might be attributed to variances in the demographic composition of the study populations, the parameters observed, and the criteria for grouping.

## The impact of hyperlipidemia on the efficacy of rotator cuff injury repair

Hyperlipidemia, characterized by elevated lipid levels, has been increasingly identified as a critical factor that correlates not only with the onset of rotator cuff tears but also with the propensity for a re-tear following surgical intervention. This multifaceted influence of hyperlipidemia was substantiated in a large-scale, rigorous study conducted by Cancienne et al. (25). Their research, encompassing an extensive cohort of 30,638 patients subjected to rotator cuff repair, unveiled a significant correlation between perioperative total cholesterol and low-density lipoprotein cholesterol levels, and the rate of revision surgery following the initial repair. This finding underscores the potential for hyperlipidemia to complicate the post-operative trajectory and recovery in patients with rotator cuff tears.

Further amplifying this perspective, Garcia’s research (14) revealed a distinct disparity in the risk of re-tear between hyperlipidemic and non-hyperlipidemic patients. The study found that, compared to their non-hyperlipidemic counterparts, patients with hyperlipidemia faced a staggering fourfold increase in the risk of re-tear following rotator cuff repair. This finding is indicative of the profound influence of lipid metabolic disorders on the integrity and healing capacity of rotator cuff tissues.

Complementing these findings, Kim’s study (26) further reinforced the role of hyperlipidemia as a significant risk factor for re-tear subsequent to rotator cuff repair. The convergence of these findings across multiple studies underscores the necessity of considering hyperlipidemia in the strategic planning of both preventive and therapeutic interventions for rotator cuff injuries.

Taking this multifactorial approach a step further, Harada et al. (27) embarked on an investigation of the risk factors for re-tear following rotator cuff repair. The study’s findings highlighted that the combination of hyperlipidemia, a tear size of ≥40 mm, and a critical shoulder angle of ≥37° collectively forecasted the highest rate of re-tear. This discovery underscores the importance of a comprehensive, multi-parametric assessment in predicting the risk of re-tear and devising more effective post-operative management strategies.

## Molecular mechanism of rotator cuff injury induced by hyperlipidemia

**Figure 1:**
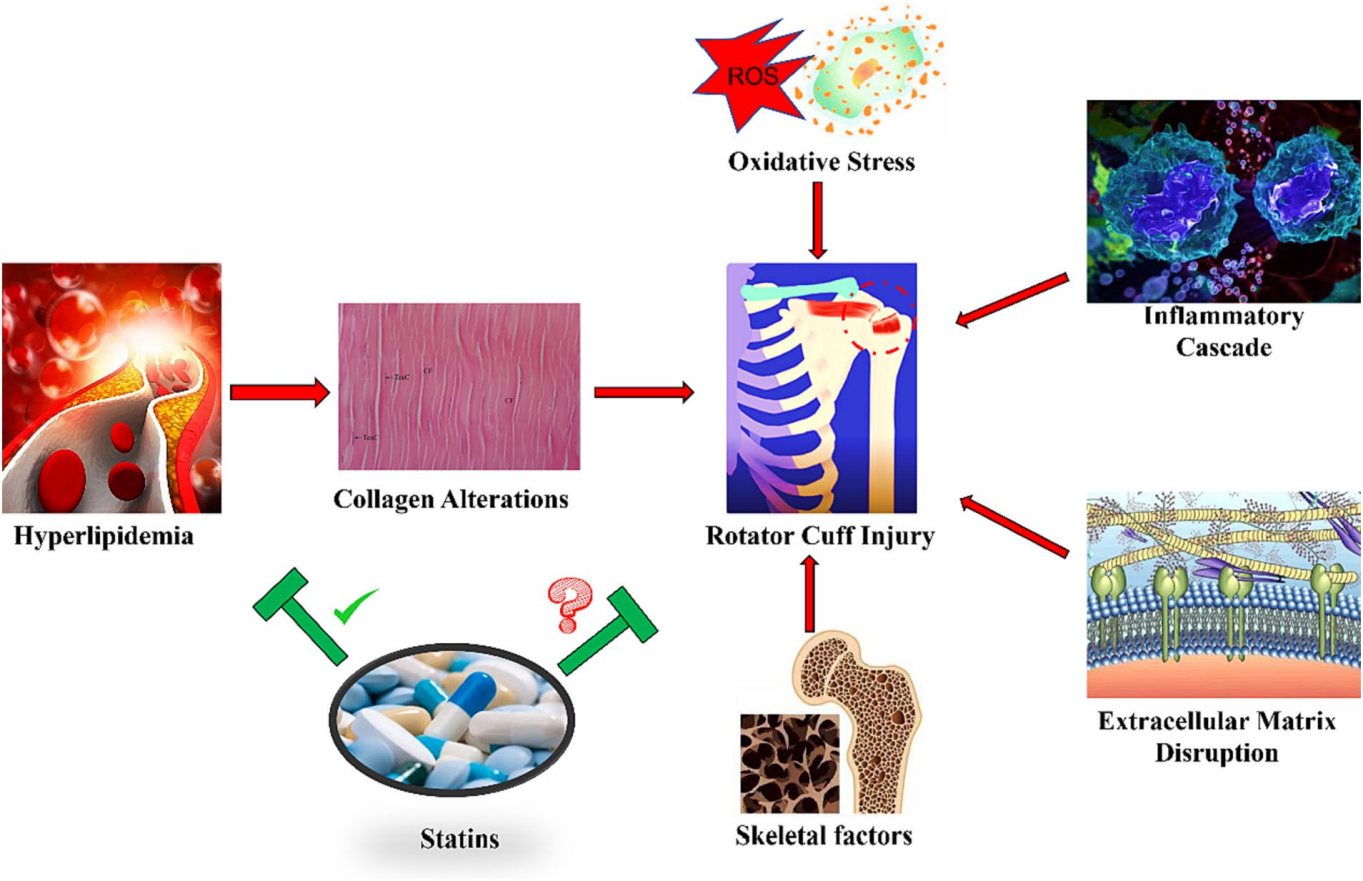
Mechanism of hyperlipidemia and statins on rotator cuff injury. The figure reflects several messages: 1. Hyperlipidemia may lead to a range of pathologic changes leading to a higher incidence of rotator cuff tears. 2. Collagen transformation, oxidative stress, inflammation, extracellular matrix disorders, and changes in the skeletal system underlie the co-morbidities of hyperlipidemia and rotator cuff injuries. 3. Statins have been clearly shown to intervene in hyperlipidemia, but the effect on rotator cuff injuries is controversial, and co-morbidities are not well understood. 4. The efficacy of statins in rotator cuff injury is controversial, and more research is needed to support the efficacy of statins in this co-morbid condition.

The intricate role of hyperlipidemia in the pathogenesis of rotator cuff injury remains somewhat nebulous, but current research illuminates a series of molecular mechanisms that may underpin the deleterious effects of this metabolic disorder on tendon health (Figure 1).

### Oxidative stress

A multitude of studies affirm the primacy of oxidative stress in triggering tendon degeneration, fibrosis, and adhesion (28, 29). Hyperlipidemia, particularly characterized by high cholesterol, can suppress the expression of tendon-related genes in tendon stem/progenitor cells via the reactive oxygen species (ROS)-activated nuclear factor kappa-B (NF-κB) signaling pathway (30, 31). Concurrently, it can instigate cell apoptosis and autophagy through the ROS-activated AKT/FOXO1 signaling pathway in these cells, culminating in dysfunction and ultimately precipitating tendon degeneration and tendinopathy (32, 33).

### Collagen alterations

Normal tendons are predominantly constituted of type I collagen, interspersed with a minor proportion of type III collagen. Aberrant cholesterol deposition in tendon tissues can provoke alterations in collagen synthesis and organization, thereby inducing structural and biomechanical alterations within the tendon matrix (34).

### Extracellular matrix disruption

The extracellular matrix (ECM) plays a pivotal role in maintaining tendon homeostasis (35). A hyperlipidemic milieu can induce changes in critical components of the ECM in tendon tissues and cells. The mechanism may involve low-density lipoprotein cholesterol (LDL-C) inducing dysfunction of tendon cells, contributing to alterations in the ECM components (36).

### Inflammatory cascade

The inflammatory microenvironment is a key player in tendon degenerative damage (37–39), and hyperlipidemia has been closely linked to systemic inflammation (40). Cholesterol molecules can activate the NF-κB signaling pathway, augmenting the expression of pro-inflammatory cytokines such as tumor necrosis factor α (TNF-α), interleukin 6 (IL-6) (41–43).

### Skeletal factors

Hyperlipidemia can exert a dual detrimental impact on bone health by inhibiting bone formation and accelerating bone resorption, thereby promoting the onset of osteoporosis (44). Notably, osteoporosis has been identified as an independent risk factor for both the genesis of rotator cuff tears and re-tear following surgical repair (45, 46). This underscores the intersection of metabolic, inflammatory, and skeletal factors in the pathogenesis and prognosis of rotator cuff injuries.

## Application of lipid-lowering drugs in rotator cuff injuries and repair

Statins, scientifically known as 3-hydroxy-3-methylglutaryl-coenzyme A (HMG-CoA) reductase inhibitors, represent the principal lipid-lowering agents in clinical practice (47). By inhibiting HMG-CoA reductase, the rate-limiting enzyme in cholesterol synthesis, statins effectively curtail cholesterol production. This, in turn, upregulates the expression of LDL receptors on the cell surface, thereby accelerating the metabolic clearance of serum LDL. However, the potential influence of statins on rotator cuff injuries and their recovery trajectory engenders a degree of controversy in the current literature.

Several studies have elucidated a beneficial role for statins in the context of rotator cuff injuries and their post-injury repair. Dolkart et al. (48) employed a rat model of rotator cuff tear to probe the impact of atorvastatin on tendon-bone healing following rotator cuff repair surgery. The experimental findings suggested that atorvastatin could activate the COX2/PGE2/EP4 pathways, thereby fostering tendon cell proliferation, migration, and differentiation, and ultimately bolstering the biomechanical integrity of the tendon-bone interface. Parallel findings were obtained in an animal study conducted by Hao et al. (49), demonstrating that silk protein fortified with simvastatin could stimulate osteogenic differentiation of bone marrow mesenchymal stem cells and collagen synthesis via the β-catenin signaling pathway, thereby enhancing tendon-bone interface healing. Lin et al. (15) conducted an 11-year longitudinal study corroborating that hyperlipidemia constitutes a risk factor for rotator cuff injuries, and statin therapy in hyperlipidemic patients could mitigate this risk. A clinical study by Cancienne et al. (25) revealed a positive correlation between perioperative lipid levels and the rate of revision surgery following rotator cuff repair, suggesting that statin therapy could potentially decrease this revision rate. Similarly, a recent case–control study by Lee et al. (50) incorporated 104 hyperlipidemic patients who underwent arthroscopic rotator cuff repair, among whom 66 individuals were in the statin group and 38 in the non-statin group. The follow-up findings indicated a lower re-tear rate in the statin group.

Conversely, several studies have reported potentially detrimental effects of statins on tendon health. Animal experiments conducted by Oliveira et al. (51, 52) demonstrated that statins could disrupt collagen fiber organization and alter tendon matrix composition, thereby diminishing the biomechanical resilience of the tendon and increasing the risk of rupture. Kaleağasıoğlu et al. (53) discerned that statins could precipitate tendon calcification, which could in turn compromise its biomechanical properties. Animal experiments and clinical cohort studies by Eliasson et al. (54, 55) showed that statins could suppress tendon cell proliferation and elicit deleterious effects on tendon collagen and matrix. Furthermore, some studies found that statins did not improve the prognosis following rotator cuff repair surgery. In a study conducted by Amit et al. (1), 77 hyperlipidemic patients underwent arthroscopic rotator cuff repair, among whom 38 individuals were on statins and 39 were not. The follow-up found no significant differences in shoulder joint function, postoperative fatty infiltration, and re-tear rates between the two groups. Similarly, a study by Zeng et al. (56) found no significant difference in postoperative shoulder function scores between hyperlipidemic patients undergoing arthroscopic rotator cuff repair who were or were not on statin therapy perioperatively.

Potential reasons for the varied outcomes observed across different studies may be due to different baseline population data, different administered doses, and different treatment times, etc. Finally, it should be mentioned that there are still some limitations in the literature cited in this current review. (1) Most of the clinical studies have a low grade of evidence. (2) Animal studies are not sufficiently mechanistically insightful. (3) There is no direct evidence for the effect of statins on co-morbidities, and more clinical and basic experimental evidence is needed to support it.

## Summary and perspectives

The significance of hyperlipidemia in the context of rotator cuff injuries is progressively gaining momentum in medical research. The intricate interplay between hyperlipidemia and these injuries remains somewhat enigmatic, necessitating large-scale, multicentric prospective studies to conclusively ascertain whether hyperlipidemia augments the susceptibility to such injuries. The molecular pathways through which hyperlipidemia instigates rotator cuff injuries are yet to be fully elucidated, thereby requiring a more comprehensive exploration through animal models and cellular experiments.

Moreover, the influence of statins on rotator cuff injuries and the subsequent healing of the tendon-bone interface post-surgery continues to be a subject of debate (57). The impact of other widely used lipid-lowering therapeutics, such as ezetimibe and probucol, as well as novel lipid-lowering agents, on tendon health remains nebulous and warrants a more thorough investigation.

The unraveling of the mechanistic role of hyperlipidemia in rotator cuff diseases, coupled with a deeper understanding of the potential therapeutic benefits of lipid-lowering drugs, could potentially offer transformative insights into the etiological basis and clinical management of rotator cuff injuries. Such insights could pave the way for more personalized and effective therapeutic regimens, enhancing the prognosis for patients with these injuries.

## Author contributions

YQ: Investigation, Methodology, Project administration, Resources, Writing – original draft, Writing – review & editing. HH: Conceptualization, Data curation, Investigation, Writing – original draft, Writing – review & editing. RW: Investigation, Methodology, Visualization, Writing – original draft, Writing – review & editing. YZ: Conceptualization, Data curation, Formal analysis, Writing – original draft. XF: Data curation, Formal analysis, Writing – original draft. FX: Funding acquisition, Supervision, Validation, Writing – review & editing. ZL: Conceptualization, Supervision, Validation, Visualization, Writing – original draft, Writing – review & editing. QW: Conceptualization, Funding acquisition, Resources, Supervision, Validation, Visualization, Writing – original draft.
